# The effect of cooperative learning on nursing students' creative thinking disposition and self-regulated learning

**DOI:** 10.3389/fpsyg.2026.1828201

**Published:** 2026-06-15

**Authors:** Dilek Erden

**Affiliations:** Department of Nursing, Faculty of Health Sciences, Tekirdağ Namık Kemal University, Tekirdağ, Türkiye

**Keywords:** cooperative learning, creative thinking, nursing, nursing education, self-regulated learning

## Abstract

**Aim:**

This study aimed to determine the effect of a nursing education course based on the cooperative learning model on the creative thinking disposition and self-regulated learning skills of students preparing to provide patient education.

**Methods:**

This was a quasi-experimental study with a one-group pretest-post-test design. The study sample included 91 third-year students enrolled in the Education in Nursing course during the 2022–2023 academic year. Data were collected before and after the education session using a Student Information Form, the Self-regulated Learning Scale in Clinical Nursing Practice (SRLSCNP), and the Marmara Creative Thinking Dispositions Scale (MCTDS).

**Results:**

Compared to pre-education values, the students' post-education creative thinking dispositions and MCTDS sub-dimensions of self-discipline, innovation search, courage, and inquisition were higher. In addition, students' self-regulated learning levels and the motivation sub-dimension were also higher. The study revealed positive relationships between the pre-training flexibility subscale and motivation (*r* = 0.518; *p* < 0.001), the self-regulated learning scale (*r* = 0.682; *p* < 0.001), and learning strategies (*r* = 0.717; *p* < 0.001); and positive relationships between post-training innovation search and the self-regulated learning scale (*r* = 0.521; *p* < 0.001), learning strategies (*r*: 0.533; *p* < 0.001), and motivation (*r*: 0.357; *p* = 0.001).

Upon course completion, a statistically significant positive relationship was found between the learning strategies and the self-discipline sub-dimension.

**Conclusions:**

The nursing education course, designed to prepare students for the implementation of patient education and based on a cooperative learning model, contributed to the development of students' creative thinking skills and their ability to learn independently.

## 1 Introduction

The educational role of nurses encompasses planned educational practices aimed at protecting and improving individual, family, and community health, promoting recovery in cases of illness, and fostering appropriate health behaviors (Açikgöz and Baykal, 2023). Patient education is one of the primary nursing roles. Patient education enables patients to manage their illness by participating in decisions about their own healthcare (Kroon et al., 2025). The most important aspect of the patient education process is the active involvement of the patient in the education process. Effective patient education enables patients to gain awareness about their care and treatment process and actively participate in decision-making processes (Jawwad et al., 2025). To achieve this, healthcare professionals can be equipped with sufficient knowledge, attitudes, and skills during their undergraduate education. Healthcare professionals equipped with these skills can learn how to plan and implement an accurate and effective patient education process tailored to patients' needs, enabling patients to actively participate in their own care and treatment process.

Nursing education is the process of providing students with cognitive, affective, and psychomotor skills to attain professional competence. Educators often benefit from cooperative learning models that put students at the center of the education process. Cooperative learning is a process in which students take an active role in a group to achieve common program objectives, taking responsibility for their education (Demiray et al., 2020; McWilliams et al., 2021). As a concept, cooperative learning provides group interaction in which participants share work to fulfill a specific task or responsibility (Mägi et al., 2024). Students experience more discussions and higher-level learning during this process than with individual learning. The goal of cooperative learning is for each student to become stronger on their own as a result of the collaborative learning process (Johnson et al., 2007).

Five elements stand out in the formation of the collaborative learning process: (1) Positive commitment: all group members complete their responsibilities by working together and providing support to maximize their learning (Johnson et al., 2007). Group members who are given different tasks and have different roles develop positive dependency. Group members believe that they can be successful when other members are successful and work together to fulfill the assigned tasks (McWilliams et al., 2021; Bayrakçeken et al., 2015). (2) Individual responsibility: group members fulfill their individual responsibilities in a cooperative learning environment. While personal responsibility constitutes part of the student's performance, group activities comprise the majority (Bayrakçeken et al., 2015). (3) Encouraging interaction: group members encourage each other to achieve the goals they set by supporting, providing feedback, and influencing each other (Johnson et al., 2007). (4) Social skills: skills such as critical thinking, self-confidence, active listening, empathy, and building personal relationships are acquired in cooperative learning environments (Johnson et al., 2007). (5) Group processing: groups discuss how to achieve their objectives in the learning process and maintain effective working relationships. Discussions simplify the learning process, eliminate inappropriate actions, and enable students to develop and improve their ability to work as part of a group. The process ends with a celebration of the performance of the group members (Johnson et al., 2007).

Many studies have shown that cooperative learning is an effective model for improving students' problem-solving, decision-making, and communication skills, as well as increasing academic performance (Darabi et al., 2024; Tinungki et al., 2024; Keramati and Gillies, 2023). Cooperative learning allows students to gain motivation and deep learning during the education process (Keramati and Gillies, 2023). In addition, it enables students to develop creativity skills and increase professional competence (Molintas et al., 2023). Collaborating on work in an education program based on the cooperative learning model allows for the generation of innovative ideas and solutions and nurtures creativity. In group work, students with different views and experiences develop new ways of thinking through brainstorming, thereby promoting their creativity and developing creative thinking skills. Thus, collaborative learning environments encourage innovation and enable students who can think creatively to succeed professionally (Wei, 2023). Moin et al. (2024) conducted a study using the cooperative learning method with medical students. The participants reported that learning became more interesting, and their creativity improved in collaborative learning environments.

Group work is a hallmark of cooperative learning; however, it is actually a learning environment where individual learning is also achieved and students must take individual responsibility for their assignments, which increases their motivation in the process. Defining individual responsibilities within group work is extremely important in designing collaborative environments (Johnson et al., 2007; Rivera Pérez et al., 2020). At this point, self-regulated learning emerges. Self-regulated learning is defined as the individual's ability to take responsibility for their learning by adapting to the conditions in the environment, setting goals, planning, determining strategies, implementing those strategies, coping with the negativities experienced in the implementation process, adapting, monitoring the process, and performing self-evaluations (Zimmerman and Pons, 1986). Students with self-regulated learning skills make advancements in motivational domains by developing higher self-efficacy in their learning process, in behavioral domains by making environmental arrangements for better learning, and in metacognitive domains by planning, monitoring, and evaluating the methods of accessing information. In other words, self-directed learning refers to the use of cognitive, affective, and behavioral characteristics necessary for the achievement of goals (Delibalta and Taşdelen Teker, 2024; Keçeci, 2017). Students develop these traits by taking responsibility for their learning and achieve self-regulated learning in cooperative learning environments (Haataja et al., 2022). Self-regulated learning is important in higher education as university students with self-regulated learning skills may be able to perform better academically and be more responsible for time and study management. Therefore, it is critical that higher education institution's structure educational programs to develop these self-regulated learning skills (Theobald, 2021).

Few studies have been conducted on how educational programs based on the cooperative learning model are implemented in nursing education and their effects on nursing students (McWilliams et al., 2021; Keramati and Gillies, 2023; Ilaslan and Demiray, 2022; Wang et al., 2021; Zhang and Chen, 2020). At the same time, one of the primary roles of nurses is that of educator. Nurses provide patient education during hospital admission, the care process, and the discharge process. Therefore, it is extremely important for nursing students to learn the patient education process. For this reason, this study aimed to determine the effect of a nursing education course based on the cooperative learning model on the creative thinking disposition and self-regulated learning skills of students preparing to provide patient education.

The research questions investigated were as follows:

1. How does a nursing education course focused on patient education based on cooperative learning affect students' creative thinking tendencies?
2. How does a nursing education course focused on patient education based on cooperative learning affect students' self-regulated learning skills?
3. Is there a relationship between the creative thinking tendencies and self-regulated learning skills of nursing students?

## 2 Methods

### 2.1 Aim

This study aimed to determine the effect of a nursing education course based on the cooperative learning model on the creative thinking disposition and self-regulated learning skills of students preparing to provide patient education.

### 2.2 Design

This was a quasi-experimental study with a one-group, pretest-post test control design. The reporting of the study was conducted under the guidance of Transparent Reporting of Evaluations with Nonrandomized Designs (Des Jarlais et al., 2004). The study was registered as a clinical trial. Clinical trials registration number: NCT06033443.

### 2.3 Participants

The study population consisted of third-year nursing students studying at the Faculty of Health Sciences, Department of Nursing, which provides four-year undergraduate nursing education at a state university in Turkey (*N* = 112). In the research, sample width was calculated using the G^*^Power V. 3.1.9.6 (Kiel University, Germany) program. With the power analysis of the students forming the sample population, (α) = 0.05 and (1 – β) = 0.95, the effect size was calculated as 0.478. The minimum number of samples was determined as 49. Considering the possibility of data loss, the sample included third-year nursing students who enrolled in the Education in Nursing course in the 2022–2023 academic year and completed all data collection tools of the study (*n* = 91).

### 2.4 Interventions

Following obtaining ethics committee approval, institutional permission, and individual consent, data were collected between October 2022 and March 2023. Prior to the start of the course, the researcher utilized a student information form. Students who met the inclusion criteria were informed about the study, their consent was obtained, and the Self-regulated Learning Scale in Clinical Nursing Practice (SRLSCNP) and the Marmara Creative Thinking Dispositions Scale (MCTDS) were administered face-to-face. The education program based on the cooperative learning model within the scope of the nursing education course was given face-to-face for 14 weeks, with 4 h of theoretical knowledge per week and 4 h of practice per week. An E-book, “*Patient Education Guide*,” was prepared to introduce the education program. The guide included the stages of the patient education plan, as well as an introduction to the cooperative learning model and how the cooperative learning process was to be managed during the implementation process. A meeting was held with the participating students, during which the education program based on the cooperative learning model was introduced. At the meeting, heterogeneous groups of six students were formed. Each group was informed that they would prepare a patient education plan related to the subject they determined by making observations during the implementation process.

The education program was designed based on five elements of cooperative learning:

1. Positive commitment: students achieved the learning outcomes together in line with the patient education plan they prepared and were aware that an individual group member's initiatives affected other members. Students were informed accordingly, and the educator guided the groups throughout the education process.
2. Individual responsibility: tasks such as preparing a patient education plan based on needs, determining the aim and objective of the education, preparing teaching materials specific to the target group, and evaluating the education were allocated to each group member during the education process. Group members took individual responsibility and did their part because this responsibility affected the group's performance. The educator supervised the students' roles in cooperative groups in practice lessons. Each student's in-group responsibilities were determined, and the educator guided them.
3. Encouraging interaction: all group members encouraged and motivated each other to achieve individual and group goals. The educator encouraged students to cooperate and develop different ideas by entering into discussion environments throughout the process.
4. Social skills: group members interacted with each other by sharing their ideas and discussing group activities. Students were guided to interact with each other by performing group work in practice lessons.
5. Group processing: group members discussed and evaluated their working order, corrected negative situations experienced during the working process by intervening, and maintained positive situations. During the education process, the educator observed the working order of the group members, and negative situations experienced by the students in acting together were resolved by the group members under the educator's guidance. The theoretical lessons were conducted using the educator-centered lecture method along with presentations. In addition, brainstorming and question-and-answer sessions were conducted. The units were visualized with concept maps. In practice courses, students practiced for the first 7 weeks, preparing a patient education plan with collaborative group work. The students conducted group work, brainstorming, and discussions. In the last 7 weeks of the practice course, all group members presented the patient education plans they had prepared in the classroom with collaborative group work, using presentations, role-play, moulage studies, educational videos created by themselves, simple models, brochures, and posters. Table 1 shows the educational content and methods used.

**Table 1:** Content and methods used in cooperative learning education program.

Theoretical courses			Intervention
Week	Session content	Session methods	
1	Basic concepts and functions of education	Theoretical lecture Question and answer Discussion	Introducing cooperative learning education using the Guide to Education in Patient, Creation of cooperative learning groups of 6 students each, Sharing cooperative learning steps with group members, determining roles and responsibilities, Implementing the education program within the framework of cooperative learning elements (positive commitment, individual responsibility, encouraging communication, social skills, group processing)
2	Determination of learning needs	Theoretical lecture Question and answer		
3	Planning and programming of training	Theoretical lecture Question and answer Brainstorming Concept map		
4	Teaching methods and techniques	Theoretical lecture Question and answer Concept map Discussion		
5	Instructional technologies and materials	Theoretical lecture Brainstorming Question and answer		
6	Organizing the learning environment	Theoretical lecture Brainstorming Question and answer		
7	Measurement and evaluation in education	Theoretical lecture Question and answer Concept map		
8–14	Presentations of cooperative learning Group work		Group work, brainstorming, Discussion environments Student presentations Role-play, Demonstration with simple models, Preparing training videos, Creating concept maps Moulage studies, Preparing brochures and posters

The procedure for developing a patient education plan consists of the following stages: identifying the target audience, determining learning needs, defining the purpose of the education, setting educational objectives, selecting the instructional methods and techniques to be used during the education process, designing the instructional materials to be used during the education process, implementing the education, and evaluating the education. Each group developed a patient education plan by following these steps. The students also learned the steps involved in preparing a patient education plan in sequence during their weekly classes and structured the process of developing the plan step by step through cooperative group work. They then presented the patient education plans they had prepared during class sessions through collaborative group activities, using presentations, role-playing, model-based exercises, educational videos they created themselves, simple models, brochures, and posters. In this way, the intervention group activities were conducted over a 14-week period. Group activities were evaluated based on the following criteria: identification of the target audience, determination of learning needs, definition of the training objective, setting of training goals, selection of appropriate teaching methods and techniques to be applied during the training process, design of appropriate and creative teaching materials for the training process, implementation of the training, and criteria for evaluating the training. Educators and students subjectively evaluated each of the above criteria as adequate, in need of improvement, and inadequate.

*After the education*, students were administered the SRLSCNP and the MCTDS.

### 2.5 Data collection

The faculty member responsible for running the course collected the study data using the Student Information Form, the SRLSCNP, and the MCTDS. In the introduction of the course, the students were informed about the purpose and process of the research, that participation was voluntary, that their personal information would not be used elsewhere, and that they should use pseudonyms when filling out the forms. In addition, they were assured that participating in the study would not affect their course grade. The data collection tools distributed to the students, who were given 45 min to complete them, were administered before (Week 1) and after the course (Week 14).

#### 2.5.1 Student information form

The Student Information Form, developed by the researcher based on the literature to determine socio-demographic characteristics, included nine questions about the students' sociodemographic characteristics and their thoughts about being creative.

#### 2.5.2 Self-regulated learning scale in clinical nursing practice (SRLSCNP)

The SRLS-CNP developed by Baysan and Orgun (Baysan and Orgun, 2023) consists of 16 items under two sub-dimensions: motivation (seven items) and learning strategies (nine items). It is a 5-point Likert-type scale on which responses are given between 1 = Strongly disagree and 5 = Strongly agree. No item is reverse scored. The minimum possible score is 16, and the maximum score is 80. Cronbach's alpha internal consistency coefficient for the overall reliability of the scale was reported as 0.89 (Baysan and Orgun, 2023). In the present study, Cronbach's alpha coefficient of the scale was 0.84.

#### 2.5.3 Marmara creative thinking dispositions scale (MCTDS)

The MCTDS developed by Özgenel and Çetin (Özgenel and Çetin, 2017) consists of 25 items under six sub-dimensions: self-discipline (five items), innovation search (eight items), courage (four items), inquisition (three items), doubt (two items), and flexibility (three items). It is a 5-point Likert-type scale on which responses are given between 1 = Never and 5 = Always. There are no reverse-scored items in the scale. The minimum score possible is 25, and the maximum score is 125. Cronbach's alpha internal consistency coefficient for the overall reliability of the scale was reported as 0.87 (Özgenel and Çetin, 2017). In the present study, Cronbach's alpha internal consistency coefficient of the scale was 0.90.

### 2.6 Data analysis

The data were analyzed using IBM SPSS V23 (IBM Corp., Armonk, NY, ABD) statistical software. The normality of the data was assessed using the Kolmogorov-Smirnov and Shapiro–Wilk tests. The dependent samples *t*-test was used to compare data showing a normal distribution between two groups. The Wilcoxon test was used to compare data that did not follow a normal distribution. To examine the relationship between continuous parameters that did and did not follow a normal distribution, the Spearman rho and Pearson correlation coefficients were used, respectively. The effect size has been calculated (Cohen's *d*, paired correlation). Analysis results were presented as frequency (percentage) for categorical variables, and as mean ± standard deviation and median (minimum–maximum) for quantitative variables. The significance level was considered to be *p* < 0.050.

### 2.7 Ethical considerations

The study was conducted following the principles of the Declaration of Helsinki. Approval was obtained from the institution where the study would be conducted and the university's Non-Interventional Clinical Research Ethics Committee (Number: 2022.188.10.12) before starting. Participation in the study was voluntary, and informed consent was obtained from each student before the study was initiated. The students were informed that the data collected would be used only for study purposes and that their confidentiality would be protected. They were not asked for any data that would reveal their identity information.

## 3 Results

### 3.1 Descriptive characteristics of the students

Of the participating students, 65.9% were female, the mean age was 21.29 ± 2.23 years, and their weighted grade point average was 3.07 ± 0.27. In addition, 96.7% of the students were single, and 70.3% stayed in student dormitories. While 67% of the students thought they were creative before their education, 93.4% considered themselves creative after their education (Table 2).

**Table 2:** Description of sociodemographic information (*n* = 91).

Variables	*X* ±SD	Median (min–max)
Age (years)	21.29 ± 2.23	21 (19–40)
Grade point average	3.07 ± 0.27	3.05 (2.49–3.65)
	*n*	*%*
Sex		
Male	31	34.1
Female	60	65.9
High school graduated from		
Anatolian high school	67	73.6
General high school	5	5.5
Science high school	7	7.7
Health vocational high school	11	12.1
Technical high school	1	1.1
Marital status		
Single	88	96.7
Married	3	3.3
Residence		
Home with family	10	11
Home with friends	9	9.9
Home alone	8	8.8
Student dormitory	64	70.3
Pretest: do you think you are creative?		
Yes	61	67
No	30	33
Posttest: do you think you are creative		
Yes	85	93.4
No	6	6.6

### 3.2 Comparison of the pretest-posttest scores of the SRLSCNP and MCTDS

Table 3 details and compares the total scores of the students' SRLSCNP and MCTDS before and after the education. Accordingly, a significant difference was found between self-discipline (*T* = −7.981; *p* < 0.001; ES = 0.591; % 95 GA [0.439: 0.711]), innovation search (*T* = −6.065; *p* < 0.001; ES = 0.449; % 95 GA [0.268: 0.600]), courage (*T* = −4.268; *p* < 0.001; ES = 0.315; % 95 GA [0.117: 0.489]), inquisition (*T* = −8.278; *p* < 0.00; ES = 0.610; % 95 GA [0.463: 0.725]), and the total score of the MCTDS (*T* = −11.702; *p* < 0.001; ES = 1.227; % 95 GA [0.951: 1.502]). However, no significant difference was observed in the doubt (*T* = −1.067; *p* = 0.286; ES = 0.077; % 95 GA [−0.131: 0.279]) and flexibility (*T* = −1.391; *p* = 0.164; ES = 0.093; % 95 GA [−0.116: 0.293]) sub-dimensions of the scale after the education.

**Table 3:** Comparison of the pretest-posttest SRLSCNP and the MCTDS scores.

	Pretest	Posttest		Test statistics		*p*	ES [% 95 GA]
Scales	*X* ±SD	Median (min–max)	*X* ±SD	Median (min–max)			
Motivation	25.13 ± 3.95	25 (12–35)	31.23 ± 2.99	32 (23–35)	−7.623	< 0.001^*^	0.564 [0.405: 0.690]
Learning strategies	38.57 ± 5.12	39 (26–45)	39.62 ± 4.04	40 (29–45)	−1.536	0.124	0.114 [−0.094: 0.312]
SRLSCNP	63.70 ± 8.37	66 (38–80)	70.85 ± 6.12	71 (57–80)	−5.7	< 0.001^*^	0.422 [0.237: 0.578]
Self-discipline	3.14 ± 0.56	3.2 (1.8–5)	4.25 ± 0.48	4.20 (3–5)	−7.981	< 0.001^*^	0.591 [0.439: 0.711]
Innovation search	3.77 ± 0.56	3.75 (1.88–5)	4.29 ± 0.48	4.25 (3.13–5)	−6.065	< 0.001^*^	0.449 [0.268: 0.600]
Courage	3.71 ± 0.72	3.75 (2–5)	4.12 ± 0.58	4.25 (3–5)	−4.268	< 0.001^*^	0.315 [0.117: 0.489]
Inquisition	3.10 ± 0.41	3 (2–4)	4.48 ± 0.45	4.67 (3–5)	−8.278	< 0.001^*^	0.610 [0.463: 0.725]
Doubt	4.23 ± 0.67	4.5 (2–5)	4.30 ± 0.57	4.50 (2–5)	−1.067	0.286^*^	0.077 [−0.131: 0.279]
Flexibility	4.18 ± 0.78	4.33 (1.67–5)	4.32 ± 0.51	4.33 (3–5)	−1.391	0.164^*^	0.093 [−0.116: 0.293]
MCTDS	3.64 ± 0.44	3.6 (2.04–4.68)	4.28 ± 0.41	4.28 (3.24–5)	−11.702	< 0.001^**^	1.227 [0.951: 1.502]

^*^Wilcoxon test; ^**^Dependent Samples *t*-test; ES [95% CI] = effect size [95% confidence Interval]: ^*^Paired correlation (*r*) and ^**^Cohen's *d* (*d*).

When the data of the self-regulated learning scale before and after the education were compared, a significant difference was found between motivation (*T* = −7.623; *p* < 0.001; ES = 0.564; % 95 GA [0.405: 0.690]) and the total score of the scale (*T* = −5.7; *p* < 0.001; ES = 0.422; % 95 GA [0.237: 0.578]). However, no significant difference was observed in the learning strategy (*T* = −1.536; *p* = 0.124; ES = 0.114; % 95 GA [−0.094: 0.312]) subscale.

### 3.3 Pretest–posttest comparison of the relationship between the SRLSCNP and MCTDS scores

Examination of the correlations among the pre-education variables showed that there was a significant and positive correlation between motivation, a sub-dimension of the SRLS-CNP, and self-discipline (*r* = 0.211; *p* = 0.044), innovation search (*r* = 0.489; *p* < 0.001), courage (*r* = 0.397; *p* < 0.001), inquisition (*r* = 0.251; *p* = 0.016), doubt (*r* = 0.435; *p* < 0.001), flexibility (*r* = 0.518; *p* < 0.001) and total MCTDS score (*r* = 0.557; *p* < 0.001). There was a statistically significant positive correlation between learning strategies, a sub-dimension of the SRLSCNP, and innovation search (*r* = 0.597; *p* < 0.001), courage (*r* = 0.532; *p* < 0.001), inquisition (*r* = 0.216; *p* = 0.04), doubt (*r* = 0.698; *p* < 0.001), flexibility (*r* = 0.717; *p* < 0.001) and MCTDS total score (*r* = 0.708; *p* < 0.001). However, there was no statistically significant correlation between the learning strategies and the self-discipline sub-dimensions (*p* = 0.074). In addition, there was a statistically significant positive correlation between the total score of the SRLSCNP and self-discipline (*r* = 0.214; *p* = 0.042), innovation search (*r* = 0.599; *p* < 0.001), courage (*r* = 0.502; *p* < 0.001), curiosity (*r* = 0.252; *p* = 0.016), doubt (*r* = 0.646; *p* < 0.001), flexibility (*r* = 0.682; *p* < 0.001) and total score of the MCTDS (*r* = 0.707; *p* < 0.001). Although these correlations remained similar after the education, a statistically significant positive relationship was found between the learning strategies and the self-discipline sub-dimensions (*r* = 0.447; *p* < 0.001; Table 4).

**Table 4:** Pretest-posttest comparison of the relationship between SRLSCNP and MCTDS scores.

Scales	Motivation		Learning strategies		SRLSCNP total	
	*r*	*p*	r^*^	*p*	r^*^	*p*
Pretest						
Self-discipline	0.211^*^	**0.044**	0.188	0.074	0.214	**0.042**
Innovation search	0.489^*^	**< 0.001**	0.597	**< 0.001**	0.599	**< 0.001**
Courage	0.397^*^	**< 0.001**	0.532	**< 0.001**	0.502	**< 0.001**
Inquisition	0.251^*^	**0.016**	0.216	**0.040**	0.252	**0.016**
Doubt	0.435^*^	**< 0.001**	0.698	**< 0.001**	0.646	**< 0.001**
Flexibility	0.518^*^	**< 0.001**	0.717	**< 0.001**	0.682	**< 0.001**
MCTDS total	0.557^**^	**< 0.001**	0.708	**< 0.001**	0.707	**< 0.001**
Posttest						
Self-discipline	0.272	**0.009**	0.447	**< 0.001**	0.436	**< 0.001**
Innovation search	0.357	**0.001**	0.533	**< 0.001**	0.521	**< 0.001**
Courage	0.283	**0.006**	0.419	**< 0.001**	0.406	**< 0.001**
Inquisition	0.333	**0.001**	0.519	**< 0.001**	0.517	**< 0.001**
Doubt	0.297	**0.004**	0.405	**< 0.001**	0.406	**< 0.001**
Flexibility	0.229	**0.029**	0.315	**0.002**	0.307	**0.003**
MCTDS total	0.355	**0.001**	0.550	**< 0.001**	0.536	**< 0.001**

*r*: Spearman's rho correlation coefficient; *p*: Pearson's correlation coefficient; ^*^Spearman's rho correlation coefficient; ^**^Pearson's correlation coefficient. Confidence intervals are reported at the 95% level (The significance level was considered to be *p* < 0.050).

## 4 Discussion

Today, training nurses who can think creatively, take responsibility for their learning development, and manage this process through self-regulated learning is undoubtedly very valuable. This study examined whether a 14-week educational course based on the collaborative learning method influenced nursing students' creative thinking tendencies and levels of self-regulated learning, and discussed the application of interactive, innovative learning approaches, such as the cooperative learning model, in nursing education. When interpreting effect sizes, a value of ES ≥ 0.30 is considered significant (Valentine and Cooper, 2003). Since the tendency toward creative thinking (ES = 1.22) in this study had a significant effect size, cooperative learning environments may contribute to nursing students' dispositions toward creative thinking. Furthermore, inquisition (ES = 0.610), self-discipline (ES = 0.591), innovation search (ES = 0.449), and courage (ES = 0.315) also exhibit significant effect sizes. Research in the literature indicates that nursing students who receive education based on a cooperative learning model exhibit increased inquisition, feel more encouraged by faculty support, and demonstrate positive developments in their attitudes toward creative thinking in the search for innovation (Keramati and Gillies, 2023; Zhang and Chen, 2020; Özgenel and Çetin, 2017; Keramati and Gillies, 2024). However, the effect size of the self-discipline subscale is statistically significant. Self-discipline is the ability to stay committed to one's goals and overcome obstacles to achieve them. Through self- discipline, students can take control of their learning during the educational process by engaging in deeper learning and further develop their professional competencies (Chan et al., 2023). Increased self-discipline among nursing students can contribute to the development of professional competencies. In this study, motivation (ES = 0.564) and the level of self-regulated learning (ES = 0.422) among nursing students had a significant effect size. Studies involving students in cooperative learning environments have shown that students' motivation increases, and they demonstrate greater effort in learning (Moin et al., 2024; Keramati and Gillies, 2024; Hu, 2023; Redondo-Rodríguez et al., 2023). By helping one another with their studies, students take ownership of their education and continue to work in small groups toward the goals they have set, thereby making their learning more purposeful and enabling them to achieve their learning objectives (Schuler et al., 2024; Najoan et al., 2024). In the present study, students were assigned specific tasks during individual and group activities as part of the cooperative learning process, and they worked on these tasks during clinical practice sessions following the theoretical instruction. In this context, this learning environment may contribute to students' levels of self-regulated learning. Studies conducted with teacher candidates and nursing students, which yielded similar results to this research, concluded that improvements in students' self-regulated learning levels were observed following the cooperative learning process (Moura et al., 2024; Sanaie et al., 2019).

The study found no relationship between learning strategies and self-discipline prior to the intervention; however, after the intervention, a statistically significant positive relationship was found between learning strategies and the subdimensions of self-discipline. Self-regulated learning refers to the process by which an individual structures their learning activities using appropriate learning strategies and coordinates cognitive control and motivational beliefs throughout the process (Knof et al., 2024). In the present study, this can be explained by the positive relationship between learning strategies and self-discipline. Nursing students can develop their self-discipline skills by overcoming obstacles and exercising self-control while developing appropriate learning strategies in cooperative learning environments (Bezerra, 2019). Harianto et al. (2020) found that cooperative learning environments encourage and enhance students' development of self-discipline. The results of the current study are similar to those mentioned earlier. Given these results, the learning strategies implemented in cooperative learning environments may contribute to improving self-discipline.

According to Dancey and Reidy, values between (+0.1 and +0.3) indicate a weak positive correlation, values between (+4 and +6) indicate a moderate positive correlation, and values between (+7 and +9) indicate a strong positive correlation (Dancey and Reidy, 2007; Akoglu, 2018). When examining the correlation values in the highest range prior to the study, there was a moderate positive correlation between the flexibility subscale and motivation (*r*: 0.518) and the self-regulated learning scale (*r*: 0.682), and a strong positive correlation with learning strategies (*r*: 0.717). Flexibility refers to the ability to adapt to a situation by trying different ways of thinking (Özgenel and Çetin, 2017). When examining its relationship with motivation, studies in literature indicate a connection between motivation, flexibility, and creativity. These studies indicate that the flexibility variable supports adaptation to the learning strategies implemented in the learning environment and to motivation (Akçay and Döş, 2026; Toprak et al., 2024). Another study also noted a positive correlation between flexibility and self-regulated learning among students who participated in an online learning program (Demir, 2024). The finding of significant relationships among flexibility, motivation, learning strategies, and self-regulated learning prior to the training suggests that students felt the need to adapt to the situation and be effective in the current context because they were faced with a new learning environment at the beginning of the training process.

When examining the correlation coefficients in the highest range following the training, it is notable that there is a moderate positive correlation between innovation search and self-regulated learning (*r* = 0.521) as well as learning strategies (*r* = 0.533), and a weak positive correlation with motivation (*r* = 0.357). Creativity and innovation enable students to think differently and uniquely, while also supporting their active participation in the learning process. Motivation is extremely valuable in the learning environment as it initiates and sustains these behaviors (Avci and Durak, 2023). In their study conducted with university students, Avci and Durak (2023) reported that motivated students scored higher on all subdimensions of the creative thinking dispositions scale. In another study, it was reported that the development of self-regulated learning skills positively contributes to students' creativity (Gundogan, 2025). Using innovative learning strategies in nursing education can greatly support nurses' ability to contribute to community health and wellbeing. This is because a nursing student who neglects creative thinking and innovation search, and who argues that existing care is sufficient, may focus less on individual patient needs and contribute minimally to the decision-making process (Silva et al., 2025). The emergence of meaningful relationships between the innovation search and motivation, learning strategies, and self-regulated learning following the training suggests that the program may have supported not only students' adaptive skills but also their tendencies to explore, try alternative methods, and seek out new knowledge. This situation suggests that while learners may initially focus on adaptation during the learning process, as their education progresses, these traits give way to more active, inquisitive, and innovation-oriented characteristics. Consequently, students who are eager to keep up with innovations, seek out new ideas, and are motivated by their work can contribute more actively to the learning process. As the nurses of the future, these students can play a more active role in patient care and decision-making processes.

## 5 Conclusions

There is evidence of improvements in the creative thinking tendencies and self-regulated learning skills of nursing students enrolled in an educational program based on the cooperative learning model. In light of these findings, active learning models and methods, such as cooperative learning, can be incorporated into the curriculum to enhance students' self-regulated learning and creative thinking skills. Another result obtained from the study revealed a significant positive relationship between learning strategies and self-discipline. The ability of nursing students to determine their learning strategies in the learning process and to take responsibility for their learning process can increase their professional competency by improving self-discipline. After training, the study found a positive correlation between the students' search for innovation search and their self-regulated learning. They can use different creative learning methods, taking into account the characteristics and needs of the target audience in the patient education process. This can help patients manage their own care and treatment processes more effectively.

## 6 Limitations and suggestions for future research

While this study provides insights into the effects of collaborative learning on creative learning and self-regulated learning, it has certain limitations. First, the data are based on participants' self-reports. Consequently, response bias may occur, which could lead to an overestimation of the intervention's effect. It is recommended that future studies include randomized controlled trials with intervention and control groups, qualitative interviews regarding student experiences, and research incorporating performance-based assessments. Second, this is a quasi-experimental study conducted exclusively with the intervention group. The reason for the absence of a control group is that the study was conducted over a 14-week academic term. During this period, students engaged in cooperative learning activities in both a clinical hospital setting and a classroom setting. However, students interact with one another in both clinical practice settings and theoretical learning environments. Given that students in the intervention and control groups may interact more frequently with one another, which could influence the results, the study was designed as a quasi-experimental study. The use of randomized controlled or controlled quasi-experimental designs is also recommended for future studies. Third, the study was conducted at a single institution in Türkiye, which may have resulted in a homogeneous sample structure in areas such as the curriculum, educational resources, and educational policies. This homogeneity may limit the generalizability of the study by restricting the diversity of the data. It is recommended that future studies be conducted in different regions and across multiple institutions. Fourth, the study was conducted over a 14-week period, and no long-term follow-up was conducted afterward. Longitudinal follow-up studies could be conducted to monitor students' creative thinking and self-regulated learning skills and make improvements as needed. Fifth, the study focuses on examining students' creative thinking and self-regulated learning skills. Other studies that implement cooperative learning may focus on academic performance or clinical skill assessments.

## Data Availability

The data supporting the findings of this study are not publicly available due to ethical and privacy considerations but are available from the corresponding author upon reasonable request.
